# Supplementary material for: Post-operative bracing following adult spine deformity surgery: Results from the AO Spine surveillance of post-operative management of patients with adult spine deformity
Source: PLoS One. 2024 Apr 16;19(4):e0297541. doi: 10.1371/journal.pone.0297541 (PMC11020681; doi:10.1371/journal.pone.0297541)
Supplement: Attachment — Submitted filename: authors response.docx [file pone.0297541.s001.docx]

**The authors’ responses to the reviewers’ comments**

We are hereby submitting our revised manuscript (Post-operative bracing following adult spine deformity surgery: results from the AO Spine surveillance of post-operative management of patients with adult spine deformity). We have revised the manuscript following the instructions of the reviewers. The details are as below:

To Reviewers #1 and #3:

Thank you very much for your support. Your thought full comments were very much appreciated.

To Reviewer #2:

Thank you very much for your important questions and suggestions. All corrections were made as per your suggestions. Here are our replies to your comments.

1. Abstract:
   -How many surgeons were surveyed? It would be helpful to know the response rate.
   Methods:
   -280 out of 6000 is a very low response rate. And of those only 116 matched the inclusion criteria.

Thank you for your suggestion. Since this e-mail based survey was sent out to the e-mail addresses of global AO Spine members, the accurate number of the participants who received the invitation could not be reported due to the potential duplications. However, we do know that the survey was sent to more than 6000 surgeons and this could be used as a denominator. Thus, the assumed response rate should be between 4 to 5%. The low response rate was certainly one of the limitations of the present study and this was added in the limitation section.

*Given the complexity of the questionnaire and the absence of financial rebates, we consider approximately 5% in the present survey to be reasonable. However, there was a potential risk of response bias while the response rate alone does not determine the validity of the results.*

2. Introduction:
-What is the correlation between PJK, pseudoarthrosis and postoperative bracing for ASD surgery? To the best of my knowledge, there is none. In fact, there is no evidence supporting postoperative bracing in this context. You state as much in your second paragraph, but the interesting question is why do surgeons still prescribe bracing without evidence of efficacy?

Thank you for the comment. As you stated, there is no solid evidence to support the brace usage in terms of complication prevention. Two additional studies were cited in this context. The reason why the surgeons still prescribe bracing is exactly what we wanted to elucidated in the present study and a sentence to put an emphasis on this point was added.

*Therefore, it is worthwhile to understand the reasons behind brace prescriptions among surgeons managing ASD, despite the lack of supporting evidence for the prevention of complications.*

*6.	Lord EL, Ayres E, Woo D, Vasquez-Montes D, Parekh Y, Jain D, et al. The Impact of Global Alignment and Proportion Score and Bracing on Proximal Junctional Kyphosis in Adult Spinal Deformity. Global Spine J. 2023;13:651-8.*

*7.	Pizones J, Boissiere L, Moreno-Manzanaro L, Vila-Casademunt A, Perez-Grueso FJS, Sanchez-Marquez JM, et al. Does the use of postoperative brace help preventing mechanical complications following adult deformity surgery? Eur Spine J. 2023;32:914-25.*

Methods:
-280 out of 6000 is a very low response rate. And of those only 116 matched the inclusion criteria.

Thank you for the comment. The low response rate is certainly unfortunate and we added this issue as one of the key limitations of the study.

*First, the response rate was relatively low unfortunately. We needed to focus on the surgeons who manage ASD on a regular basis to capture the real-world trend, which further inevitably limit the number of valid responses. Given the complexity of the questionnaire and the absence of financial rebates, we consider approximately 5% in the present survey to be reasonable. However, there was a potential risk of response bias, while the response rate alone does not determine the validity of the results.*

-For the question "the main purpose for bracing" asked in your survey, was this a free-form response? Or did you have prefilled answer choices for the survey participants?

Thank you for your question. The response options were prepared both as prefilled choices and a free-form. The detail description was added.

*For questions 2) and 3), several pre-filled response options were provided along with a free-form option, allowing individuals to provide individual statements. Multiple selections were permitted.*

-A question for "why do you not use a brace?" would have been interesting.

Thank you for your suggestion. This was unfortunately out of scope of our current investigation, but it certainly has the value to understand why some surgeons are actually avoid bracing. This issue was added as a limitation.

*Thirdly, while we have made an effort to investigate the practices of surgeons who prescribe braces, it would also be meaningful to understand the rationales from those who oppose brace prescription. Braces have been associated with downsides such as cost and skin irritation.*

Results:
-What does it mean to use bracing selectively? What is the indication for "selective" brace use?

Thank you for the question. Some surgeons answered that they “occasionally” prescribe bracing. It is understandable to use the brace only for who the surgeons might think were high-risk for complications. Unfortunately, the present study did not reveal the detailed indication for which these “selective” user prescribed the brace.

*Among them, 38 surgeons (33%) only occasionally prescribed the brace for selected cases, with the details of the indications unrevealed.*

-Results don't appear particularly profound

The present study was a descriptive paper to show the real-world data collected by an on-line survey. Instead of the statistical analyses, for which we normally do in the scientific comparative study, we rather focused on the accurate description of the current trend among the spinal surgeons with regard to brace prescription. The results were surprisingly diverse and we strongly believe that the present paper will serve as a foundation for further research planning and development to elucidate the efficacy of bracing in adult spinal deformity surgery. However, as per your suggestion, in order to strengthen the granularity of the data, we added the short description of the responses made in a free-form format.

*Among the free-form responses (15%), some surgeons highlighted motives associated with patients' emotions, such as providing reassurance or serving as a reminder for precaution.*

*Secondly, responses to questions regarding indications and influencing factors may have been potentially biased, as we provided several options for frequently encountered responses to be chosen, while open-ended responses were also accepted in a free-form format.*

Discussion:
-Should include a limitations section

Thank you for your suggestions. All the issues raised above were clearly explained as limitations in the discussion section.

Please do not hesitate to contact us if there are some problems in the revised manuscript. Thank you very much for your kind cooperation in advance.
